# mTOR is a Key Protein Involved in the Metabolic Effects of Simple Sugars

**DOI:** 10.3390/ijms20051117

**Published:** 2019-03-05

**Authors:** Gemma Sangüesa, Núria Roglans, Miguel Baena, Ana Magdalena Velázquez, Juan Carlos Laguna, Marta Alegret

**Affiliations:** 1 Department of Pharmacology, Toxicology and Therapeutic Chemistry, School of Pharmacy and Food Science, University of Barcelona, 08028 Barcelona, Spain; gemmasanguesa@gmail.com (G.S.); roglans@ub.edu (N.R.); mbaena@uic.es (M.B.); avelazquezpy@gmail.com (A.M.V.); jclagunae@ub.edu (J.C.L.); 2 Institute of Biomedicine, University of Barcelona, 08028 Barcelona, Spain; 3 Centro de Investigación Biomédica en Red de Fisiopatología de la Obesidad y Nutrición (CIBERObn), 28029 Madrid, Spain

**Keywords:** mTOR, fructose, glucose, liver, lipid metabolism, gluconeogenesis

## Abstract

One of the most important threats to global human health is the increasing incidences of metabolic pathologies (including obesity, type 2 diabetes and non-alcoholic fatty liver disease), which is paralleled by increasing consumptions of hypercaloric diets enriched in simple sugars. The challenge is to identify the metabolic pathways affected by the excessive consumption of these dietary components when they are consumed in excess, to unravel the molecular mechanisms leading to metabolic pathologies and identify novel therapeutic targets to manage them. Mechanistic (mammalian) target of rapamycin (mTOR) has emerged as one of the key molecular nodes that integrate extracellular signals, such as energy status and nutrient availability, to trigger cell responses that could lead to the above-mentioned diseases through the regulation of lipid and glucose metabolism. By activating mTOR signalling, excessive consumption of simple sugars (such as fructose and glucose), could modulate hepatic gluconeogenesis, lipogenesis and fatty acid uptake and catabolism and thus lipid deposition in the liver. In the present review we will discuss some of the most recent studies showing the central role of mTOR in the metabolic effects of excessive simple sugar consumption.

## 1. Introduction

**Figure 1:**
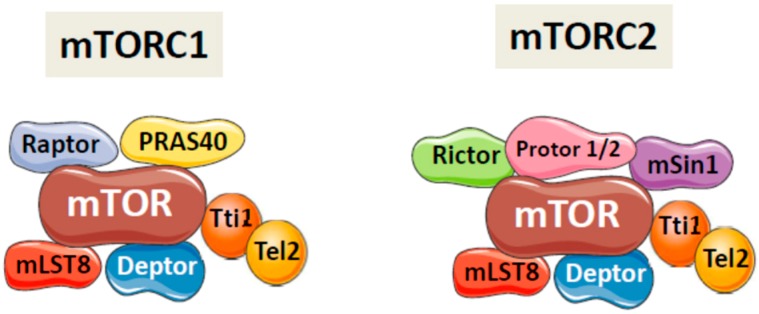
Components of mTORC1 and mTORC2 complexes. mTORC1 and mTORC2 share the core proteins mTOR and mammalian lethal with SEC13 protein 8 (mLST8), the Tti1/Tel2 complex and the inhibitory protein DEP domain-containing mTOR-interacting protein (DEPTOR). In addition, mTORC1 contains regulatory-associated protein of mTOR (Raptor) and the inhibitory subunit proline-rich Akt substrate of 40 kDa (PRAS40), whereas mTORC2 contains rapamycin-insensitive companion of mTOR (Rictor) and the regulatory proteins Protor1/2 and mSin1.

Mechanistic (formerly mammalian) target of rapamycin (mTOR) is a serine/threonine protein kinase that forms the catalytic centre of two multi-protein complexes termed mTORC1 and mTORC2, which have different compositions and responses to upstream signals [1,2]. As it is shown in Figure 1, both complexes share the core proteins mTOR and mammalian lethal with SEC13 protein 8 (mLST8), the Tti1/Tel2 complex and the inhibitory protein DEP domain-containing mTOR-interacting protein (DEPTOR). The mTORC1 complex contains regulatory-associated protein of mTOR (Raptor) and the inhibitory subunit proline-rich Akt substrate of 40 kDa (PRAS40), whereas mTORC2 contains rapamycin-insensitive companion of mTOR (Rictor) and the regulatory proteins Protor1/2 and mSin1. Both complexes are activated by insulin and related growth factors, such as insulin-like growth factors, while mTORC1 can also be activated by nutrients (amino acids, cholesterol and simple sugars), oxygen and the cellular energy status (sensed via ATP levels) [1,3].

The downstream effects of mTORC1 and mTORC2 activity (reviewed in Reference [4]) are also different. Briefly, mTORC1 activation induces cellular growth through: (i) activation of mRNA translation, driven by the phosphorylation of the ribosomal protein S6 kinases (S6K1/2), which activate several substrates associated with the initiation of mRNA translation and the phosphorylation of eIF4E binding proteins (4EBP), which release factor eIF4E to enable the formation of the translation initiation complex; (ii) promotion of metabolic effects including increased de novo lipid synthesis via the activation of sterol response element-binding protein (SREBP) and a shift to glycolysis instead of oxidative phosphorylation; and (iii) inhibition of catabolic pathways such as autophagy, mainly by the phosphorylation of UNC-51-like kinase 1 (ULK-1). By contrast, mTORC2 activation promotes cell survival and proliferation through the phosphorylation of several kinases including Akt, which is one of the main transducers of insulin signalling.

In summary, the role of mTOR complexes is to coordinate cell responses to energy availability by promoting or repressing anabolic and catabolic molecular pathways in response to different stimuli, including nutrients and growth factors. This review focuses on mTOR regulation by a specific type of nutrients (simple sugars) and its consequences on lipid and carbohydrate metabolism, aiming to gain insight into the mechanisms by which an excessive intake of simple sugars causes metabolic diseases such as dyslipidaemia, diabetes and non-alcoholic fatty liver disease (NAFLD).

## 2. Regulation of mTORC1 Activity by Simple Sugars

**Figure 2:**
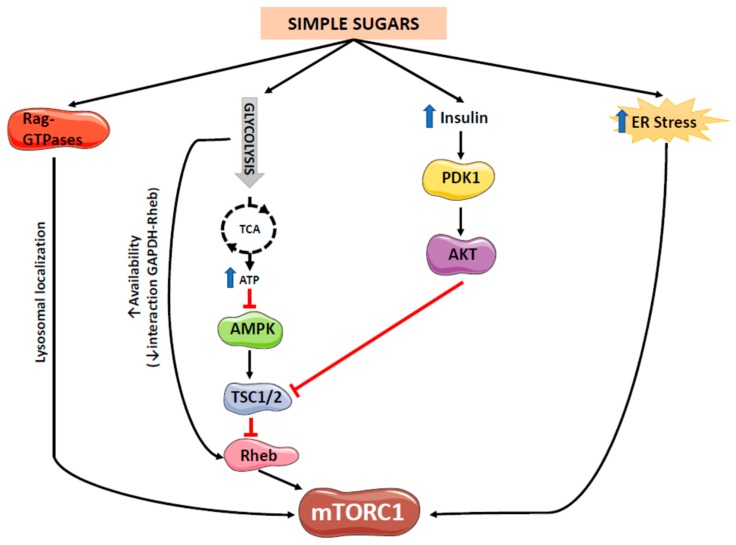
Proposed mechanisms of mTORC1 activity regulation by simple sugars. mTORC1 activation by simple sugars might be mediated by several mechanisms, including (i) Rag-GTPases activation, which cause mTORC1 lysosomal localization, (ii) increased glycolytic flux, which might increase Rheb availability and inhibit AMPK activity, (iii) increased plasma insulin levels and (iv) increased ER stress.

Of the two mTOR complexes, only mTORC1 seems to be regulated by simple sugars (Figure 2). Direct regulation of mTORC1 activity by carbohydrates is less well known than that by amino acids. Activation of mTORC1 by amino acids is initiated by the stimulation of the Rag family of GTPases, which recruits mTORC1 to the outer lysosomal membrane [2]. Using mice that constitutively express an active form of RagA, Efeyan et al. showed that mTORC1 activation by carbohydrates also involves the Rag-GTPases [5,6]. This shared mechanism between amino acids and carbohydrates does not directly activate mTORC1 but causes its lysosomal localization, where the Ras-homolog enriched in brain (Rheb) GTPase resides and activates mTORC1 by promoting mTOR kinase activity. mTOR phosphorylates several substrates, including its autophosphorylation at Ser-2481, which indicates mTOR-specific catalytic activity [7].

In studies conducted by our group in female rats supplemented with 10% (*w*/*v*) liquid fructose for different periods of time (two weeks, two months and seven months), we consistently showed a marked increase in hepatic mTOR phosphorylation at Ser-2481 [8,9,10]. As Rheb overexpression has been reported to promote the phosphorylation of mTOR at this position [11], we explored whether fructose induced mTOR phosphorylation by increasing Rheb expression. We did not detect a significant increase in the hepatic levels of Rheb protein in female rats supplemented with liquid fructose for two months [8]. A cell-based model of mTORC1 regulation by glucose proposed that high glucose levels increased glycolytic flux and reduced the interaction of Rheb with GAPDH, thus increasing the availability of Rheb to interact with and activate mTORC1 [12]. Our co-immunoprecipitation experiments showed that this was not the mechanism for fructose-induced mTORC1 activation, since Rheb-GAPDH and Rheb-mTOR interactions were not affected in the livers of fructose-supplemented rats [8].

Carbohydrates also regulate mTORC1 indirectly via adenosine monophosphate-activated protein kinase (AMPK), which senses the fluctuations in energy levels due to glucose availability. AMPK is another cellular energy sensor that is activated by increased AMP/ATP ratio caused by either reduced ATP production or excessive ATP consumption [13]. AMPK has the opposite role to that of the mTOR system. While mTOR is activated by high energy availability and promotes anabolic routes to use this energy in cell growth and proliferation, AMPK senses low energy statuses, for example glucose deprivation and promotes catabolic processes to obtain energy. Glucose deprivation activates AMPK not only by increasing the AMP/ATP ratio but also by depletion of the glucose metabolite fructose-1,6-biphosphate. Reduced levels of this metabolite facilitate the formation of an AMPK-containing protein complex in the lysosomal membrane, which stimulates the kinase LKB1 to phosphorylate and activate AMPK [14]. AMPK activation can inhibit the mTORC1 pathway through two main mechanisms: phosphorylation of tuberous sclerosis complex (TSC) 2 at Ser-1387, which activates the upstream mTOR inhibitor TSC1/TSC2 complex [15,16] or direct inhibition by phosphorylating Raptor [17]. It is not yet known which of these mechanisms predominate but it has been proposed that TSC2 phosphorylation may counteract growth factor-activated mTORC1, whereas Raptor phosphorylation has been reported to inhibit basal mTORC1 activity [18].

When glucose enters the glycolytic pathway to form pyruvate for the tricarboxylic acid (TCA) cycle, ATP is generated and AMPK is inhibited, which leads to mTORC1 activation. However, before entering the glycolytic pathway, carbohydrates are phosphorylated by hexokinases in an irreversible reaction that consumes ATP. Although this process is similar for all carbohydrates, there are some differences. For example, glucose is phosphorylated first by glucokinase and after isomerization the resulting molecule is phosphorylated by phosphofructokinase, whose activity is tightly controlled by end-product inhibition (ATP and citrate) [19]. By contrast, fructose is phosphorylated by fructokinase and enters the glycolytic pathway by skipping the negative feedback control system. Moreover, fructose up-regulates fructokinase expression, thus inducing its own metabolism. Therefore, despite both fructose and glucose phosphorylation consuming ATP, this consumption is more rapid and intense for fructose, which may deplete ATP [20]. Paradoxically, this mechanism could lead to AMPK activation and, consequently, mTORC1 inhibition. Results from our own studies in female rats supplemented with simple sugars in liquid form for seven months indicated that fructose does not activate hepatic AMPK [21], suggesting that if ATP depletion does occur it may be transient and not associated with chronic fructose intake. In a recent study, Hu et al. confirmed this transient inhibitory effect of fructose: in mice sacrificed 30 min after receiving a 10% fructose or glucose solution by gavage, mTORC1 activity was inhibited by fructose but this inhibitory effect was lost in mice receiving a diet containing 60% fructose for one week [22]. In contrast to fructose, our results in female rats showed that chronic consumption of an equicaloric glucose solution activated AMPK, as demonstrated by a modest but significant 30% increase in phospho-AMPK α (Thr172) levels [21]. We attributed this effect to the increased plasma adiponectin levels observed only in rats consuming glucose [9], as the liver has been described to be one of the main targets of circulating adiponectin, which activates hepatic AMPK by phosphorylation [23].

Carbohydrates can also regulate mTORC1 activity through endoplasmic reticulum (ER) stress. The ER has a key role in cellular homeostasis by controlling the synthesis, folding and posttranslational modification of proteins. Perturbations in the ER folding capacity cause ER stress and trigger the unfolded protein response (UPR), which involves the activation of specific transmembrane proteins acting as stress sensors (activating transcription factor 6 [ATF6], inositol-requiring enzyme 1 [IRE1] and protein kinase RNA-like ER kinase [PERK]) to restore cell homeostasis by proper protein folding and reduction of the ER protein load [24]. The UPR is activated not only by the accumulation of unfolded proteins but also by other stimuli, including nutrient availability. Both deprivation and excessive levels of carbohydrates can induce ER stress, which may influence mTORC activity. Thus, glucose starvation causes energy stress by reducing ATP levels, which at least in some cell types can induce calcium efflux from the ER and activate the PERK branch of the UPR [25]. In addition, protein glycosylation occurs in the ER and, therefore, alterations in this process due to glucose depletion could perturb ER homeostasis and elicit also ER stress [18]. On the other hand, high levels of glucose stimulate ER stress in hepatic cells, as shown by higher levels of PERK phosphorylation [26]. Interestingly, the glucose-induced ER stress response in these cells is mediated by mTORC1, as specific mTORC1 inhibition by rapamycin prevents the phosphorylation of PERK and its downstream effector eIF2α [26]. ER stress can also elicit mTORC1 activation through ATF6 and the induction of the phosphoinositide 3-kinase (PI3K)-Akt axis; however, Akt and mTORC1 are inhibited under conditions of chronic ER stress [27].

Results from our own studies in female rats showed that fructose consumption activated mTORC1 independently of ER stress [8,9]. As mentioned before, we observed that fructose induced hepatic mTOR phosphorylation at Ser-2481 but the only UPR marker that was increased was phosphorylated IRE1, whereas the PERK branch of the UPR remained unaffected [8,9]. In fact, mTOR activation in renal cells has been shown to selectively induce IRE1 without affecting PERK and ATF6 [28]. In contrast to PERK activation, which leads to CHOP induction and JNK-mediated apoptosis, selective IRE1 activation might protect cells by promoting the elimination of misfolded proteins without inducing cell death. Moreover, as it was shown in hepatocyte-specific IRE1-null mice, IRE1 is required to prevent hepatic steatosis [29] and when it is chronically activated it may cause the regression of pre-existing steatosis. Accordingly, the lack of hepatic steatosis observed in rats supplemented with fructose or glucose for seven months could be attributed to the selective and chronic activation of IRE1 in their livers [9].

Interestingly, when we compared the effects of glucose and fructose supplementation in female rats, we found that fructose induced hepatic mTOR Ser-2481 phosphorylation to a greater extent than glucose and that only fructose activated downstream effectors of mTORC1 such as phosphorylated 4EBP1 [9]. The fact that fructose had a greater effect on mTORC1 than glucose could not be attributed to the amount of energy provided by the sugar supplementation, as rats consumed equicaloric amounts of glucose and fructose. Instead, this could be due to an indirect mechanism involving changes in plasma insulin levels. Insulin, after binding to its receptor, activates the PI3K-phosphoinositide-dependent kinase 1 (PDK1)-Akt pathway, leading to the phosphorylation of TSC2 at multiple sites, which inactivates the TSC2/TSC1 complex and activates Rheb and mTORC1 [2]. Our results showed that only fructose-supplemented rats displayed significant hyperinsulinemia after seven months of treatment and therefore, the lower level of mTORC1 activation observed with the equicaloric glucose supplementation could be attributed, at least in part, to a lack of effect on plasma insulin levels [9]. Another factor contributing to this differential effect is that, as commented before, only glucose supplementation activates AMPK [9], which has an inhibitory effect on mTORC1.

## 3. The Effects of mTOR on Lipid Metabolism and Modulation by Carbohydrates

**Figure 3:**
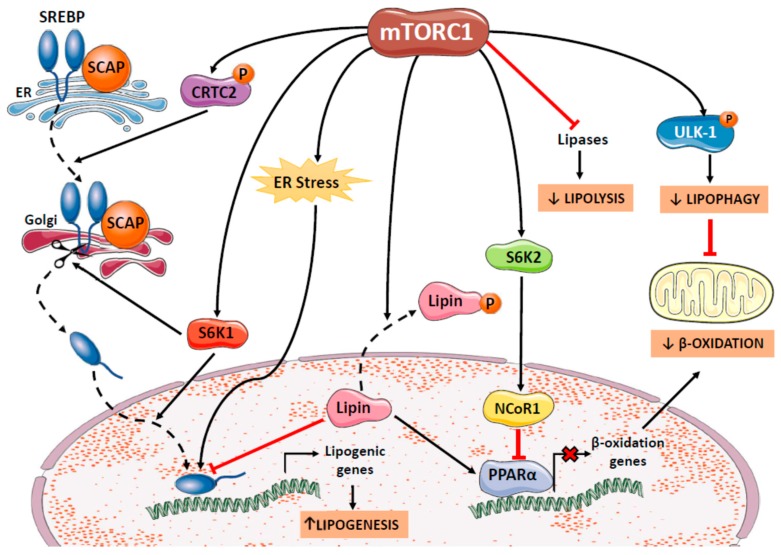
Effects of mTORC1 on lipid metabolism. mTORC1 activates lipogenesis via SREBP1c, partly through S6K1 and also by S6K1-independent mechanisms, such as the phosphorylation of CRTC2 and lipin, which causes its nuclear exclusion, and ER stress induction. In addition, mTORC1 inhibits neutral lipolysis and repress autophagy/lipophagy by phosphorylating ULK-1, which could inhibit fatty acid β-oxidation by lowering substrate availability. mTORC1 may also inhibit PPARα activity by recruiting NCoR1 to the nucleus and by inducing lipin-1 phosphorylation.

The mTOR system plays a key role in the response to nutrient abundance, in part by activating anabolic processes such as lipid synthesis. Both mTORC1 and mTORC2 can modulate lipogenesis but much more attention has been paid to mTORC1. Lipogenesis is activated by mTOR mainly through the master regulator of this process, the transcription factor SREBP1c, which belongs to the basic-helix loop-helix-leucine zipper family. SREBP1c is synthesized as a precursor that remains anchored to the ER membrane through its interaction with SREBP cleavage-activating protein (SCAP) [30]. Through a mechanism that remains unclear [31], the SCAP-SREBP1c heterodimer is transferred to the Golgi apparatus, where SREBP1c is processed by proteolytic enzymes into its mature form, that then translocates to the nucleus to induce the transcription of target genes, including those encoding the main lipogenic enzymes. The mTOR system can regulate SREBP1c activity at different levels, including transcription of its encoding gene Srebf1, processing of the precursor protein and nuclear transport of the mature protein (Figure 3) [2,31].

mTORC1 activates SREBP1c partly through S6K1 by mechanisms that have not been completely elucidated [2]. For example, a study in rat hepatocytes in which the transcriptional effects of insulin on the SREBP1c gene were eliminated showed that S6K1 is essential for SREBP1c processing but not for its transcription [32]. However, other studies have shown that S6K1 depletion in the liver of obese mice reduces the abundance of hepatic SREBP1c mRNA [33]. Moreover, a recent report showed that mTORC1-S6K1 activation may induce lipogenesis independently of SREBP1c by promoting the splicing of lipogenic mRNAs, that increases their stability [34]. In addition, mTORC1 may activate SREBP1c via S6K1-independent pathways such as by: (i) the phosphorylation of CREB-regulated transcription coactivator 2 (CRTC2), which increases the trafficking of SREBP1c to the Golgi apparatus where it is processed [35]; (ii) the phosphorylation and nuclear exclusion of lipin-1, which prevents its blocking effect on SREBP1c transcriptional activity [36]; and (iii) inducing ER stress, which may activate hepatic SREBP1c [2]. Furthermore, mTORC2 might also regulate SREBP1c, as mice with specific hepatic deletion of Rictor have been reported to show reduced SREBP1c activity and lipogenesis [37].

Thus, it could be hypothesized that simple sugars promote lipogenesis by activating the mTORC1-SREBP pathway. Our studies in rats supplemented with 10% liquid fructose (*w*/*v*) for two weeks or two months did not show increases in the nuclear active form of SREBP1c, despite higher levels of mTOR phosphorylation [8,10]. By contrast, nuclear SREBP1c levels were significantly increased in rats fed a 63% fructose in solid form for two weeks [38]. Similarly, in our chronic study in which 10% liquid sugars were supplemented for seven months, fructose but not glucose caused an increase in nuclear SREBP1c [9]. Therefore, it seems that the ability of fructose to activate SREBP1c depends on the burden of fructose to the organism, taking into account both the amount of fructose consumed and the duration of the supplementation. Moreover, the different effects of fructose and glucose on SREBP1c activity could be due to the greater effect of fructose on mTOR phosphorylation, combined with the hyperinsulinemia caused by fructose but not glucose supplementation.

mTOR affects lipid metabolism not only by inducing lipogenesis but also by inhibiting lipolysis and lipophagy (Figure 3). Both mTORC1 and mTORC2 can inhibit neutral lipolysis, a process in which cytoplasmic lipases hydrolyse cytoplasmic lipid droplets at a neutral pH [39]. The term lipophagy was coined in 2009 when it was demonstrated that the intracellular lipid content is also regulated by the lysosomal degradation of lipids through autophagy [40]. Since then, lipophagy has been reported to occur in different cell types and be essential for regulating cellular energy metabolism, as it may be activated or inhibited in response to energy requirements [41]. Lipophagy is, in fact, a specialized subtype of autophagy, using the same intracellular machinery and being regulated by the same mechanisms as those associated with autophagy, including mTORC1 [39]. Repression of autophagy by mTORC1 involves the phosphorylation of ULK-1 at Ser-757 [42]. Our studies in female rats revealed that increased phosphorylation of hepatic mTOR after fructose supplementation for two months increased phosphorylated levels of ULK-1 and led to a decrease of autophagic markers, including the LC3B-II/LC3B-I protein ratio [8]. As the inhibition of autophagy promotes hepatic triglyceride accumulation, we hypothesized that this mechanism could be responsible, at least in part, for the hepatic steatosis observed in rats sub-chronically supplemented with liquid fructose.

Finally, the mTOR system may also modulate lipid catabolism by inhibiting fatty acid β-oxidation. This could be a consequence of the inhibition of autophagy by mTORC1, as autophagy causes the breakdown of triglycerides and provides fatty acid substrates for β-oxidation. Activation of mTORC1 and inhibition of autophagy could explain why β-oxidation is inhibited in the livers of female rats after sugar supplementation for two and seven-month, without altering peroxisome proliferator-activated receptor (PPAR)α gene or protein expression [8,9]. Despite a lack of effect on PPARα expression, our results showed that the reduction of β-oxidation by fructose involved lower PPARα activity, as indicated by the reduced nuclear PPARα levels in the livers of fructose-supplemented rats [9]. This could also be related to mTORC1 activation, as mTORC1 has been reported to inhibit PPARα at least by two possible mechanisms, via nuclear receptor corepressor 1 (NCoR1) or lipin-1 [2]. NCoR1 is a transcriptional regulator that binds to several nuclear receptors. The mTORC1 substrate S6K2 has been observed to associate with NCoR1, recruiting it to the nucleus where it represses PPARα transcriptional activity [43]. In hepatic cells lipin-1 acts as a transcriptional activator of β-oxidation genes through PPARα activation. As it was previously mentioned, mTORC1 can induce the phosphorylation and nuclear exclusion of lipin-1, which could lead to the reduction of PPARα activity. There are few studies examining the effects of carbohydrates on lipin-1 in vivo. Vasiljević et al. reported that male rats supplemented with 10% liquid fructose for nine weeks showed increased microsomal levels of lipin-1 in the liver, although the extent of lipin-1 phosphorylation or its concentration in hepatic nuclear fractions were not determined [44]. Furthermore, despite the same treatment increased the nuclear contents of lipin-1 in the hearts of female (but not male) rats, this was not accompanied by increased levels of nuclear PPARα [45]. Moreover, it remains to be established whether this is a specific effect in cardiac cells and whether it is related to mTOR activity.

## 4. The Effects of mTOR on Carbohydrate Metabolism

**Figure 4:**
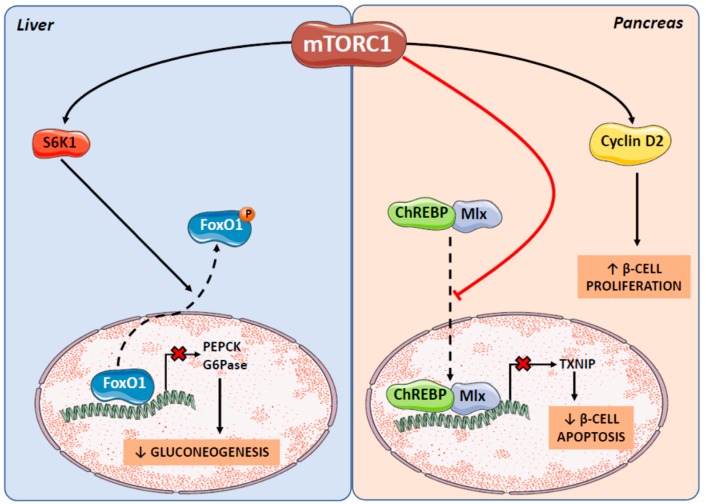
Effects of mTORC1 on carbohydrate metabolism. mTORC1 activation inhibits gluconeogenesis by promoting the phosphorylation and nuclear exclusion of FoxO1 via S6K1, which blocks its transcriptional effect on the main gluconeogenic genes PEPCK and G6Pase. Carbohydrate metabolism may be also indirectly altered by mTORC1 effects on the survival and proliferation of insulin-secreting pancreatic β-cells.

mTOR can affect carbohydrate metabolism directly, by controlling hepatic gluconeogenesis, and also indirectly, by regulating pancreatic β-cell mass and activity (Figure 4). Moreover, both mTORC1 and mTORC2 can induce insulin resistance and, thus, dysregulate glucose metabolism.

The ability of mTORC1 to control gluconeogesis was demonstrated by experiments in rapamycin-treated rats, which showed that chronic inhibition of the mTORC1-S6K1 pathway increased the hepatic expression of the key gluconeogenic genes encoding phosphoenolpyruvate carboxykinase (PEPCK) and glucose 6-phosphatase (G6Pase) in the liver [46]. The regulation of gluconeogenic gene expression is mediated partly by the transcription factor forkhead box protein O1 (FoxO1). In response to insulin, FoxO1 is phosphorylated by Akt causing its nuclear exclusion and a reduction in PEPCK and G6Pase expression. Interestingly, it has been reported that in rapamycin-treated rats, in which mTORC1 was inhibited, nuclear FoxO1 levels were increased despite hyperinsulinemia, which could account for the increased expression of PEPCK and G6Pase [46]. Although that study did not determine the extent of FoxO1 phosphorylation, Yue et al. [47] showed that the stimulation of mTOR-S6K1 signalling in the mouse hypothalamus increased FoxO1 phosphorylation, whereas treatment with rapamycin (which blocks only mTORC1 when it is administered acutely) reduced FoxO1 phosphorylation. In addition, experiments in liver-specific Rictor knockout mice demonstrated that mTORC2 also controls hepatic gluconeogenesis. Upon refeeding, these mice showed hepatic FoxO1 hypophosphorylation and nuclear localization, together with higher levels of PEPCK and G6Pase mRNA, compared to wild type mice [37]. mTORC2 phosphorylates Akt at Ser-473 and it has been shown that in mTORC2-deficient cells phosphorylation at this position is absent and FoxO1 phosphorylation is specifically reduced [48].

Thus, it seems plausible that both mTORC1 and mTORC2 activation may inhibit gluconeogenesis by promoting FoxO1 phosphorylation via S6K1 and Akt, respectively. Our results in female rats supplemented with simple sugars support this hypothesis. In rats drinking a 10% *w*/*v* fructose solution for two weeks, mTORC1 activation led to a decrease in PEPCK expression, probably via IRE1 phosphorylation that promoted the splicing of X-box-binding protein (XBP)-1, which is involved in the maintenance of glucose homeostasis [49]. Moreover, chronic glucose and fructose supplementation in rats has been reported to activate mTORC1 (shown by the phosphorylation of peroxisome proliferator-activated receptor gamma coactivator 1-alpha [PGC-1α], a direct target of S6K1 and the absence of Ser-473 Akt phosphorylation), increase FoxO1 phosphorylation and reduce the expression of PEPCK and G6Pase [9]. However, the crosstalk between mTORC1 and mTORC2 is quite complex, as cell culture experiments show that mTORC1-S6K1 signalling induce Rictor phosphorylation, with Akt and FoxO1 phosphorylation increasing when the phosphorylation position of Rictor is mutated [50]. This suggests that mTORC1 signalling could inhibit the mTORC2-Akt pathway, leading to reduced FoxO1 phosphorylation and increased gluconeogenesis.

In addition, mTOR signalling regulates the growth and proliferation of pancreatic β-cells and their ability to secrete insulin, which may also affect glucose homeostasis. Similar to the regulation of gluconeogenesis, both mTOR complexes control β-cell mass and activity, as mice deficient in S6K1 [51] or Rictor [52] exhibit reduced β-cell mass and hypoinsulinemia. The molecular mechanism underlying these effects was recently unravelled [53]. In pancreatic β-cells, mTOR interacts with a complex containing ChREBP and Max-like protein (Mlx), inhibiting its translocation to the nucleus. The ChREBP-Mlx complex regulates the transcription of thioredoxin-interacting protein (TXNIP), which is involved in the apoptosis of β-cells. Thus, the reduced nuclear translocation of ChREBP-Mlx results in reduced TXNIP expression and protects β-cells from apoptosis. Moreover, mTOR not only regulates the number of β-cells but also their specific activity, as mTOR inactivation by the overexpression of a kinase-dead mTOR mutant (which therefore affects both mTORC1 and mTORC2) leads to defective β-cell function without affecting its mass [54].

Carbohydrates, as well as other nutrients, can regulate β-cell proliferation as an adaptive response to the changes in the metabolic environment. It has been recognized for a long time that glucose regulates not only insulin secretion but also the proliferation of β-cells. However, the role of mTOR as a key player in this process has been demonstrated only recently. The proliferative effect of glucose on β-cells involves the activation of an atypical protein kinase C (PKCζ), which activates mTORC1 and subsequently induces cyclin D2 activation [55,56]. Fructose might also have a proliferative effect of on pancreatic β-cells, given the ability of fructose to activate mTORC1. However, excessive fructose consumption might be detrimental, as a high fructose diet (65% fructose in solid form) has been reported to induce pancreatic ER stress and β-cell apoptosis, which are increased when fructose is combined with a high fat diet [57].

## 5. Concluding Remarks

**Figure 5:**
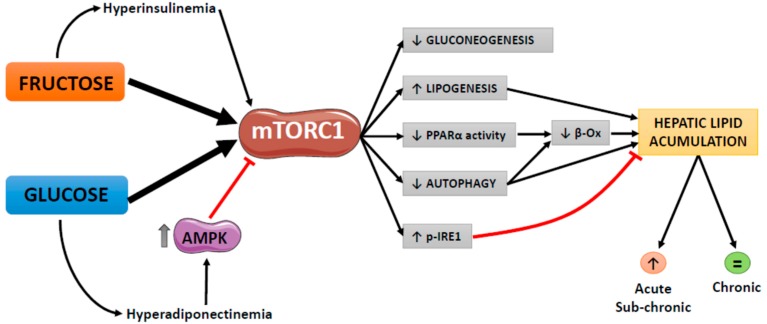
mTORC1 as a key hub transducing the metabolic effects of glucose and fructose in the liver. Fructose activates mTORC1 leading to reduced gluconeogenesis and enhanced lipogenesis which, together with PPARα/β-oxidation activity and autophagy inhibition contribute to hepatic fat deposition observed in our studies after sub-chronic administration of fructose (for two months) in female rats. By contrast, sustained activation of the mTORC1-IRE1 pathway by chronic fructose supplementation could prevent fat deposition in the liver. Fructose-induced hyperinsulinemia, which activates mTORC1 and the increase in plasma adiponectin levels caused by glucose administration, which inhibits mTORC1 via AMPK activation, lead to a different extent of mTORC1 activation by these simple sugars, which seems to be responsible for the different metabolic effects of fructose and glucose supplementation observed in our studies performed in female rats.

It is well recognized that overnutrition, together with a sedentary lifestyle, is one of the main drivers of metabolic pathologies such as fatty liver, dyslipidaemia and hyperglycaemia. However, the role of individual nutrients and the mechanisms involved have not been fully elucidated. From our studies in rats supplemented with simple sugars in liquid form (10% *w*/*v*) for different periods of time (from two weeks to seven months), we have identified hepatic mTOR, specifically mTORC1, as a key hub that transduces the signals elicited by carbohydrates to activate or inhibit molecular pathways and modulate the metabolism of lipids and carbohydrates. Thus, we have consistently observed that glucose and fructose intake increases mTOR phosphorylation and activates mTORC1. Although the specific underlying mechanism is not fully understood, we have ruled out the involvement of Rheb availability or expression and we have also demonstrated that mTORC1 activation is independent of ER stress. mTORC1 activation by glucose and fructose explains most of the metabolic effects observed in our studies in female rats, including enhanced lipogenesis and reduced gluconeogenesis (Figure 5). Moreover, mTORC1 activation might also inhibit PPARα activity and autophagy which, together with enhanced lipogenesis, contribute to hepatic fat deposition. By these mechanisms sub-chronic administration of fructose (for two months) induced hepatic steatosis in female rats. However, sustained activation of the mTORC1-IRE1 pathway by chronic fructose supplementation could prevent fat deposition in the liver. Moreover, the different extent of mTORC1 activation seems to be responsible for the different effects of fructose and glucose supplementation in rats, as only fructose-supplemented rats displayed significant hyperinsulinemia, which also activates mTORC1. On the other hand, only glucose increases plasma adiponectin levels leading to AMPK activation, which in turn inhibits mTORC1 and therefore has a weaker effect on mTORC1 activation than fructose. The mechanism by which glucose increases adiponectin levels and whether this effect is specific to rodents or may also apply to humans are yet to be established.

## Abbreviations

4EBP	eIF4E binding protein
AMPK	activating transcription factor 6
ATF6	adenosine monophosphate-activated protein kinase
CRTC2	CREB-regulated transcription coactivator 2
DEPTOR	DEP domain-containing mTOR-interacting protein
ER	endoplasmic reticulum
FoxO1	forkhead box protein O1
G6Pase	glucose 6-phosphatase
IRE1	inositol-requiring enzyme 1
mLST8	mammalian lethal with SEC13 protein 8
Mlx	Max-like protein
mTOR	mechanistic (mammalian) target of rapamycin
mTORC	mTOR complex
NAFLD	non-alcoholic fatty liver disease
NCoR1	nuclear receptor corepressor 1
PDK1	peroxisome proliferator-activated receptor
PEPCK	phosphoenolpyruvate carboxykinase
PERK	phosphoinositide 3-kinase
PGC-1α	phosphoinositide-dependent kinase 1
PI3K	proline-rich Akt substrate of 40 kDa
PPAR	protein kinase RNA-like ER kinase
PRAS40	protein S6 kinases
Raptor	rapamycin-insensitive companion of mTOR
Rheb	Ras-homolog enriched in brain
Rictor	receptor gamma coactivator 1-alpha
S6K1/2	regulatory-associated protein of mTOR
SCAP	SREBP cleavage-activating protein
SREBP	sterol response element-binding protein
TCA	thioredoxin-interacting protein
TSC	tricarboxylic acid
TXNIP	tuberous sclerosis complex
ULK-1	UNC-51-like kinase 1
UPR	unfolded protein response
XBP-1	X-box-binding protein
